# Impact of Successive Exploitation of a *Saccharomyces pastorianus* Starter Culture on Saccharide Uptake Dynamics from Wort

**DOI:** 10.17113/ftb.59.01.21.6899

**Published:** 2021-03

**Authors:** Miha Ocvirk, Nataša Kočar Mlinarič, Iztok Jože Košir

**Affiliations:** 1 Slovenian Institute of Hop Research and Brewing, C. Žalskega tabora 2, 3310 Žalec, Slovenia; 2 Pivovarna Laško Union d.o.o., Pivovarniška ulica 2, 1000 Ljubljana, Slovenia

**Keywords:** beer fermentation, saccharide metabolism, *Saccharomyces pastorianus*, glucose, fructose, maltose

## Abstract

**Research background:**

The production of lager beer includes successive repitchings of a single *Saccharomyces pastorianus* starter culture. During the beer production process, the yeast is exposed to several stress factors which could affect the fermentation performance. An incomplete fermentation represents a waste of fermentable extract and leads to a beer with higher carbohydrate levels, which could result in a beer with an atypical flavour profile. The aim of the present study is to determine the impact of successive exploitation of a single *S. pastorianus* starter culture on the wort saccharide uptake dynamics.

**Experimental approach:**

The fermentation was monitored during the production of twelve batches of beer, where the starter yeast culture was reused twelve times without any further treatment. The following beer production steps were monitored: wort production, yeast starter culture propagation, primary fermentation, secondary fermentation and the final product. The work was conducted on an industrial scale employing standard process conditions.

**Results and conclusions:**

Monitoring of the starter culture viability during successive fermentations indicated no reduction in the viability and vitality of the yeast culture. Monitoring of the fermentable wort saccharide concentrations (glucose, fructose, disaccharides and trisaccharides) revealed a correlation between an improvement in saccharide utilisation and starter culture age. Saccharide uptake efficacy proportionally matched the repitching frequency. Successive exploitation of *S. pastorianus* starter culture has a positive impact on the dynamics of saccharide utilisation from classical hopped wort and the young beer. Furthermore, the final lager beer contains no residues of fermentable saccharides that could affect the overall quality and flavour profile.

**Novelty and scientific contribution:**

Results showed the impact of twelve successive wort fermentations on the dynamics of saccharides uptake that gives brewers important information. The added value of the experiment is all the work done on the industrial scale, with control of all processes and usage of exactly the same raw materials. This study contains usable technological data on the behaviour of saccharides during brewing on the industrial scale, which is not yet found in the literature.

## INTRODUCTION

Beer production involves the following procedures: wort production, primary and secondary fermentation, filtration and packaging of the final product. The starter culture for lager production is *Saccharomyces pastorianus,* and the culture is re-used successively. The yeast must be removed in a controlled manner at the end of primary fermentation and then stored under standard conditions to preserve its quality and prevent unwanted flavour effects in the beer and also to provide the yeast for subsequent repitching (*1*). During the brewing process, the efficiency of fermentation and the character and quality of the final product are intimately linked to the amount and health of the yeast being employed. The vitality and viability of the pitched yeast contribute both to the rate of fermentation and to the organoleptic properties of the beer produced.

The number of times a yeast culture can be serially repitched is largely determined by a combination of product quality constraints and company procedures (*2*). Brewers routinely reuse cropped yeast from one fermentation to another in order to pitch subsequent fermentations. However, this practice cannot be continued indefinitely because of the increased risk of contamination and spontaneous mutations in the yeast. During brewery handling, the production strains of the yeast culture must respond positively to fluctuations in dissolved oxygen concentration, pH, osmolality, ethanol concentration, nutrient supply and fermentation temperature (*3*). The physiological condition of the pitching yeast may in turn influence the levels of organic acids, esters, higher alcohols, aldehydes and diacetyl throughout fermentation and during maturation, thereby contributing to the overall organoleptic properties of the final beer (*4*). The physical and fermentation characteristics of the working yeast culture may appear consistent, but the occurrence of genetic mutations has been reported during yeast recycling. Serial repitching extends the possibility of accumulating viable yeast variants which can compete with the original yeast culture (*5*).

Factors other than the pitching yeast starter culture can also influence the fermentation performance and beer quality; these include the wort composition and the wort aeration (oxygenation), the fermentation temperature and the size and geometry of the fermentation vessel (*6*). The wort composition influences the speed of fermentation, the attenuation degree, the amount of yeast produced and the quality of the final beer (*2*). Wort consists of simple saccharide, dextrins, amino acids, peptides, proteins, vitamins, ions (such as zinc, magnesium, manganese, calcium, sodium and potassium), nucleic acids and many other constituents too numerous to discuss in detail (*7*). While the wort composition is variable, the majority of wort share many common features. The principal carbohydrates are maltose, maltotriose, glucose, fructose and degrees of polymerisation DP4-DP10 (*8*, *9*).

During primary fermentation, wort saccharides are converted into ethanol, carbon dioxide and glycerol. Yeast biomass is also produced. The manner by which wort saccharides are utilised plays a crucial role in the final quality of the beer and determines the rate and extent of a brewing fermentation (*10*).

Glucose is the preferred fermentation substrate compared to all other wort carbohydrates. It moves across the yeast plasma membrane in a non-specific facilitated diffusion path, which modulates its affinity in response to the available glucose. Maltose is transported into the cell by the maltose permease and then hydrolysed by α-d-glucosidase. The genes for this permease and α-d-glucosidase are separately transcribed, and their expression is induced by maltose and repressed by glucose (*11*). Maltose and maltotriose utilisation is inhibited by the addition of glucose. A major limiting factor in the fermentation of wort is the repressing influence of glucose (and possibly fructose) on maltose and maltotriose uptake. Some researchers (*11*, *12*) have suggested that the inhibition of maltotriose transport by maltose is the main reason for this high maltotriose content, while others have proposed the existence of a separate maltotriose uptake system, distinct from that of maltose, in *Saccharomyces cerevisiae* (*13*).

Maltotriose, like maltose, must be internalised by the brewing yeast strain and hydrolysed into glucose molecules before it is metabolised through the glycolytic pathway. Once inside the cell, saccharides are metabolised by the Embden-Meyerhof-Parnas or glycolytic pathway to yield ethanol, carbon dioxide and glycerol as end products. In addition, a complex mixture of flavour active secondary metabolites is produced, of which the higher alcohols (or fusel) and esters are the most important. Secondary fermentation or maturation follows, when the beer is kept for an appropriate period (1–3 weeks) and at reduced temperatures (from -2 to 5 °C). At this stage, flavour maturation occurs, as well as precipitation of haze-forming materials. The residual yeast in suspension may also utilise any remaining slowly fermentable carbohydrates at this stage to generate carbon dioxide and flavour compounds, as well as to remove undesirable flavour by-products of the primary fermentation (for example, diacetyl is an important maturation marker) (*13*).

The extract in finished beer consists of approx. 85% carbohydrates (particularly in the form of the dextrins maltotetraose and maltopentaose) (*14*), 6–9% nitrogenous compounds and 0.2% glycerol (glycerine), as well as β-glucans, inorganic compounds, phenolic compounds, bitter substances, organic acids and a number of compounds which, despite their low concentrations, have an effect on the quality of the beer (*15*).

The aim of this study is to establish the effect of yeast repitching on their saccharide metabolism and the final beer quality. To our knowledge, this work represents the first example of a study at an industrial scale that used 12 successive fermentations with overall use of *S. pastorianus* TUM 34/70 as the starter culture in 3250 hL fermentation tanks. This study is therefore unique and novel, as many previous studies have been performed on the laboratory scale, where the assimilation of saccharides from wort is controlled and conditions do not directly reflect the actual stress environment encountered by yeast cultures during successive cycles on the industrial scale.

## MATERIALS AND METHODS

Important process parameters, including the fermentation rate, extract consumption, alcohol formation rate, pH value and number and viability of the yeast cells, were assessed. Industrial scale production was conducted in conical fermentation tanks (total volume 4400 hL, working volume 3250 hL). Strict traceability of the ingredients for wort production (malt, corn and hops) was assured. A conventional brewing protocol and fermentation diagram for primary and secondary fermentation was applied. After primary fermentation, the yeast culture was harvested and stored under similar conditions (*t*=4 °C, time=48 h, no mixing) prior to the next repitching cycle.

### Yeast

The yeast used in the brewing process for all 12 cycles was a lager yeast strain, *Saccharomyces pastorianus* TUM 34/70, supplied by the Yeast Centre at Weihenstephan, Freising, Germany.

### Sampling during beer production

Wort samples were taken before the starter yeast culture was inoculated (pitched) into the wort, during primary (every 24 h) and secondary fermentation (at the beginning and the end of this procedure). After beer packaging, the final products were sampled. Samples (*V*=200 mL) for saccharide determination were stored at -20 °C. All brewing quality parameters and yeast viability were analysed immediately after sampling. Samples and results were marked with successive letters, with A indicating the first fermentation cycle and L indicating the 12th fermentation cycle.

### Methods

The samples were analysed employing the methods of the European Brewing Convention (EBC). The samples were degassed by shaking for 10 min at 150 rpm on a shaker (HS 50; IKA-Werke Staufen, Germany). The samples were then filtered through a Whatman grade 597½ filter, diameter 240 mm, pore size 4–7 μm (particle retention) (Whatman, Buckinghamshire, UK). Determination of the real, original and apparent extract and original gravity were made on an SP-1m Beer Analyser (Anton Paar, Graz, Austria) instrument according to MEBAK method 2.9.6.3 (*16*). Alcohol content was determined according to the Analytica EBC method 9.4 (*17*) using SP-1m Beer Analyser (Anton Paar) instrument. The real degree of fermentation (RDF) was determined according to the Analytica-EBC method 9.5 (*18*) and calculated from the measurements of the alcohol content and real extract using the following equation:


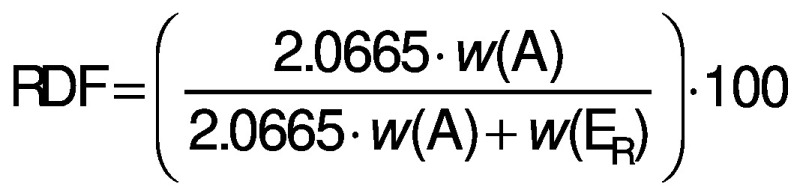


where *w*(A) is alcohol in %, *w*(E_R_) real extract in % and 2.0665 is a numerical factor.

The wort and beer pH values were determined at 20 °C according to the Analytica-EBC method 9.35 (*19*) using a pH meter (MPC227; Mettler Toledo, Urdorf, Switzerland) with an InLab Export Pro electrode (Mettler Toledo).

The viability of the yeast cells was determined by methylene blue straining according to the Analytica-Microbiologica–EBC method 3.2.1.11 (*20*), using a 40× microscope magnification (BH2; Olympus, Tokyo, Japan). The concentrations of fructose, glucose and saccharides with 2 and 3 degrees of polymerisation (DP2 and DP3) were determined by high-performance liquid chromatography (HPLC 1200 series; equipped with refractive index detector, Agilent Technologies, Santa Clara, CA, USA). The DP2 peak was primarily maltose, while DP3 peak was primarily maltotriose.

All standard compounds (glucose with 99.5% purity and fructose, maltose and maltotriose with 99.0% purity) were purchased from Fluka (St. Quentin Fallavier Cédex, France). The HPLC method cannot distinguish among different disaccharides or different trisaccharides. The saccharides in beer were detected according to the Analytica EBC method 9.27 (*21*) and in wort according to the Analytica EBC method 8.7.3 (*22*). All measurements were done in duplicates. Limit of quantification for all four tested compounds was 0.01 g/100 mL and the average relative standard deviation of the measurements was 1.0%.

### Sensory evaluation

Sensory evaluation of all beer samples included taste, odour and bitterness rating by seven experts, who gave seven independent marks. Expert panel consisted of 3 females and 4 males, aged between 32 and 56. The evaluation took place in a separate tasting room, with room temperature from 18 to 25 °C and humidity between 50 and 70%. All beer samples tasted fresh one week after the bottling. At the time of tasting, temperature of the samples was between 10 and 11 °C. Evaluation marks ranged from 1 to 5 for each parameter (data not shown).

### Statistical analysis

After sensory evaluation, OriginPro statistical software v. 2020b (*23*) was used for statistical analysis to determine average marks and their standard deviations. Sensory mark 4.5 to 5.0 means very good, pleasant, well balanced beer, a perfect example of this kind of beer, 3.5 to 4.5 means good/clean fresh beer which may have a characteristic flavour attribute at a slightly elevated or reduced level, 2.5 to 3.5 means neither good nor poor beer with off flavours at low level and/or an imbalance of brand flavour characteristics, 1.5 to 2.5 means poor beer with one or more intense off flavours and 1.0 to 1.5 means very poor beer, undrinkable/wrong product (*24*). The main purpose of sensorial evaluation was to detect possible sensory mistakes in the produced beer to be sure that all samples are adequate.

## RESULTS AND DISCUSSION

During 12 successive fermentations, 12 samples of wort, 53 samples of the primary fermentation and 24 samples of the secondary fermentation were collected. The sample amounts were sufficient to elucidate the fermentation kinetics and the yeast starter culture behaviour during prolonged exploitation under industrial conditions, which are considerably more stressful for the production yeast culture.

### Wort analyses

The original extract of wort (from A to L) was between 11.47 and 11.68% (by mass), which is within the normal mass fraction for a regular lager beer. The pH was not significantly different (Table 1). Some significantly different saccharide concentrations were observed. Wort samples E and D had lower (<6.0 g/100 mL), while wort samples G and J had the highest saccharide concentrations (>9.0 g/100 mL). However, the ratio was constant between the particular groups of fermentable wort saccharides (glucose, fructose, saccharides with DP2 and DP3). Two saccharides always showed extreme values in all 12 wort samples (from wort A to wort L). The highest value was observed for DP2 (maltose) and the lowest for fructose (Table 1).

**Table 1:** The results of the analyses of wort (from wort A to L)

Wort sample	*w*(extract)/ %	pH	*γ*(fructose)/(g/100mL)	*γ*(glucose)/(g/100 mL)	*γ*(DP2)/(g/100 mL)	*γ*(DP3)/(g/100 mL)	*γ*(overall)/(g/100 mL)
A	11.6±0.2	5.3±0.1	0.20±0.01	0.67±0.04	5.1±0.3	1.93±0.03	7.9±0.4
B	11.5±0.2	5.3±0.1	0.20±0.01	0.73±0.04	5.0±0.3	1.96±0.03	7.9±0.4
C	11.7±0.2	5.3±0.1	0.25±0.01	0.76±0.04	4.7±0.3	1.83±0.03	7.5±0.3
D	11.7±0.2	5.3±0.1	0.21±0.01	0.60±0.03	4.1±0.2	1.17±0.02	6.1±0.3
E	11.7±0.2	5.3±0.1	0.12±0.01	0.62±0.04	4.2±0.2	1.20±0.02	6.2±0.3
F	11.6±0.2	5.2±0.1	0.29±0.01	1.21±0.07	5.7±0.3	1.68±0.02	8.9±0.4
G	11.7±0.2	5.3±0.1	0.29±0.01	1.22±0.07	5.9±0.3	1.71±0.02	9.2±0.4
H	11.6±0.2	5.3±0.1	0.30±0.01	0.85±0.05	5.2±0.3	1.45±0.02	7.8±0.4
I	11.5±0.2	5.3±0.1	0.33±0.01	1.06±0.06	5.8±0.3	1.64±0.02	8.8±0.4
J	11.5±0.2	5.2±0.1	0.35±0.01	1.17±0.07	5.9±0.3	1.65±0.02	9.0±0.4
K	11.5±0.2	5.3±0.1	0.34±0.01	1.10±0.06	5.6±0.3	1.56±0.02	8.6±0.4
L	11.5±0.2	5.2±0.1	0.29±0.01	1.04±0.06	5.4±0.3	1.54±0.02	8.3±0.4

DP2 and DP3=degrees of polymerisation 2 and 3

### Primary and secondary fermentations

In total, 77 samples were collected during primary and secondary fermentations. The fermentation rates of all 12 green (immature) beer samples were comparable (82–84%). The alcohol volume fraction followed this kinetics, as expected, and was between 4.90 and 5.05%. The pH decreased from approx. pH=5.25 to 4.10. More than 50% of the pH decrease was achieved during the first 24 h of primary fermentation. The number of yeast cells during the primary fermentation increased and reached a maximum between the second and third days. The number of yeast cells in suspension subsequently decreased to approx. 13∙10^6^ cell/mL. This development was as expected at the end of the primary fermentation in order to enable proper secondary fermentation for the lager beer.

The concentration of fermentable saccharides in wort F at the end of the primary fermentation ranged from 0.29 to 0.58 g/100 mL (data not shown). The only difference was observed for the green beer samples that used yeast starter culture from wort F (1.55 g/100 mL, data not shown) and culture from wort J (1.19 g/100 mL, data not shown). The reason for this difference could be in the initial saccharide concentration in the wort (Table 1). The maximum saccharide uptake efficiency occurred during primary fermentation when yeast starter culture from wort D (92.00%) was used, and the lowest efficiency of saccharide uptake during primary fermentation occurred when the yeast starter culture from wort F was used (82.85%).

**Fig. 1:**
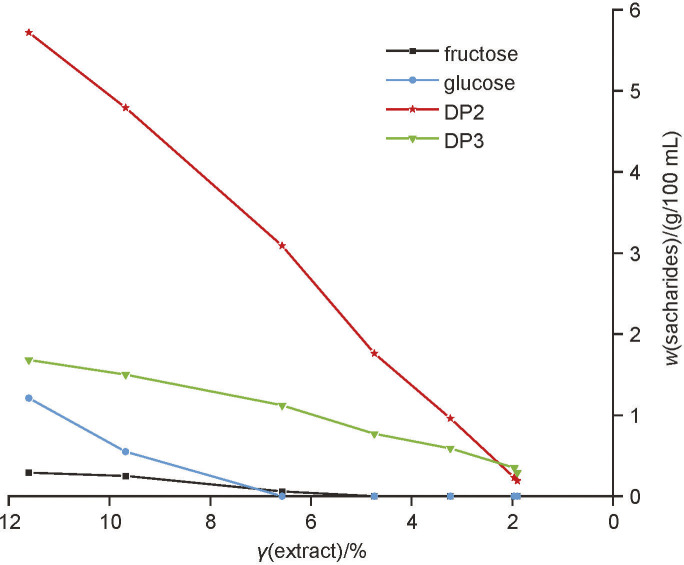
Concentrations of fructose, glucose, wort saccharides with the degrees of polymerisation 2 and 3 (DP2 and DP3) and extract during primary and secondary fermentations with the use of yeast starter culture *Saccharomyces pastorianus* (fermentation F). Standard deviation (S.D.) did not exceed 0.2

In all cases during primary fermentation, the uptake of glucose (from A to L) was completed within 48 h. Glucose was exhausted when the extract mass fraction dropped to 55% of its initial value, which is approx. 5.0%. Fructose kinetics followed that of glucose, and fructose was exhausted when the residual extract reached 3.5%. The saccharides with DP2 and DP3 were not completely exhausted during primary fermentation. However, *S. pastorianus* consumed 95% saccharides with DP2 and 76–92% with DP3 (Fig. 1).

**Fig. 2:**
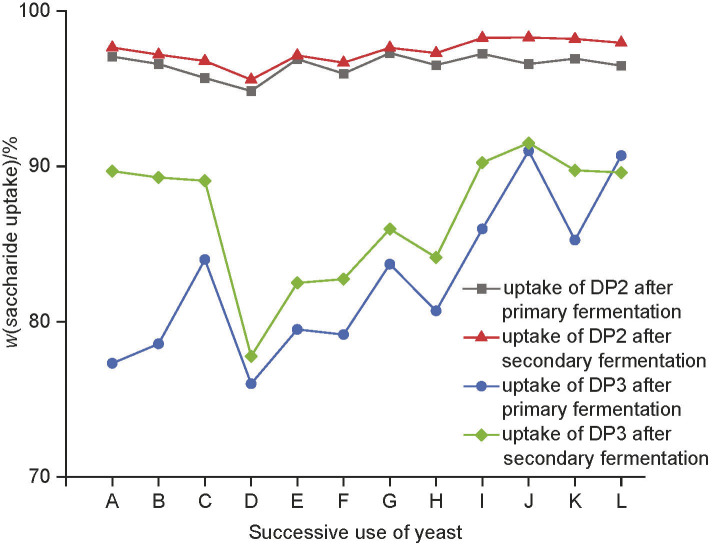
The efficiency of DP2 and DP3 saccharide uptake during primary and secondary fermentation with a successive use of starter culture *Saccharomyces pastorianus*

The monitoring of standard parameters during secondary fermentation revealed only minor changes. The alcohol volume fraction increased between 0.2 and 0.9%, depending on the number of yeast cells that remained in the green beer after racking and the wort saccharide concentration at the beginning of secondary fermentation. The maximum difference between the extract mass fraction was 0.97% (data not shown), which was detected during secondary fermentation of the green beer in wort K. During secondary fermentation, only DP2 and DP3 saccharides remained. Since most DP2 saccharides were nearly consumed during primary fermentation, only an average addition of 3% was consumed during secondary fermentation and no statistical significance was observed regarding the saccharide consumption profile. This was not the case with DP3, where up to 25% differences for DP3 consumption were observed between successive fermentations (Fig. 2).

### Yeast viability

The yeast viability was determined for the successive uses of all yeast starter cultures (from starter culture A to starter culture L). The yeast cell viability was consistent and comparable (>95%) during all successive fermentations. These results supported the conclusion that the viability of the yeast starter culture was not affected or reduced by its successive use and suggested that we could have prolonged the fermentation cycle for even further successive fermentations.

### Saccharide metabolism

Values were normalised to allow observation of the differences in the saccharide consumption dynamics by the yeast. A correlation was made individually for each saccharide. The transparency of the processed data and the efficiency uptake findings led to the formation of the following four groups: group 1: the first, second and third successive uses of the yeast starter culture (A, B and C), group 2: the fourth, fifth and sixth successive uses of yeast starter culture (D, E and F),group 3: the seventh, eighth and ninth successive uses of the yeast starter culture (G, H and I), and group 4: the tenth, eleventh and twelfth successive uses of the yeast starter culture (J, K and L).

The curves that describe the saccharide uptake dynamics during fermentation and the correlation plots fit into an exponential decay curve of the first order with the following equation:


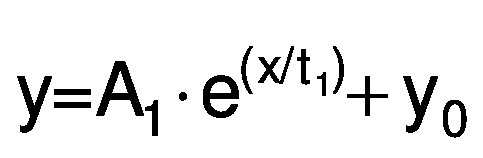


where y is the saccharide mass fraction, x is the extract mass fraction, and A_1_, t_1_ and y_0_ are constants.

### Metabolism of glucose

**Fig. 3:**
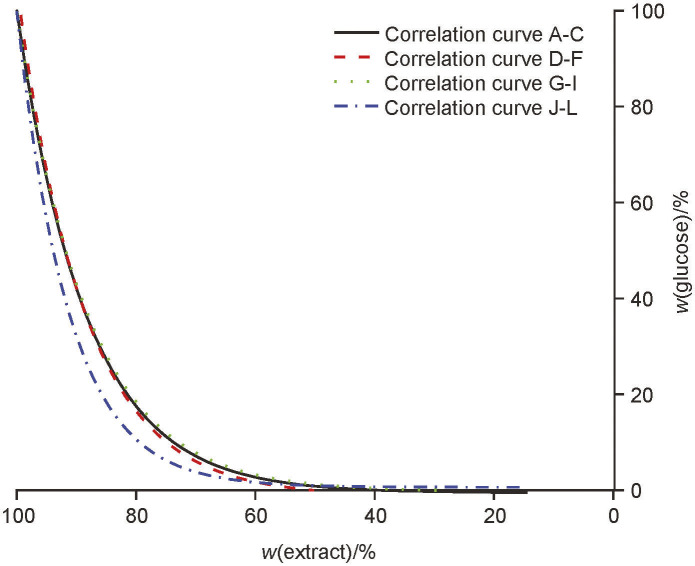
The correlation curves for glucose consumption with successive use of yeast starter culture *Saccharomyces pastorianus* for all four groups: curve A-C group 1, curve D-F group 2, curve G-I group 3 and curve J-L group 4

**Fig. S1:**
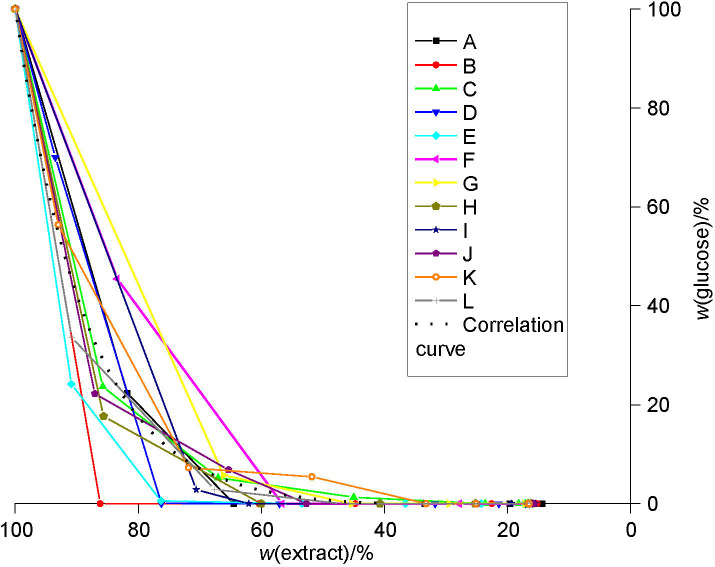
The relation between glucose and extract mass fractions during beer production with a successively used yeast starter culture (from the first (A) to the last (L) successive use)

The results of glucose consumption during beer production are shown in Fig. S1. The correlation curves for the individual groups with the differing successive use of starter *S. pastorianus* cultures are given in Fig. 3. Note that the glucose consumption was uniform and more efficient when the starter culture was used several times (*i.e.* in fermentations J, K and L [group 4]). The efficiency of the other groups in the glucose uptake did not differ significantly. As mentioned earlier, the consumption of glucose was efficient and was completed during primary fermentation.

### Metabolism of fructose

**Fig. 4:**
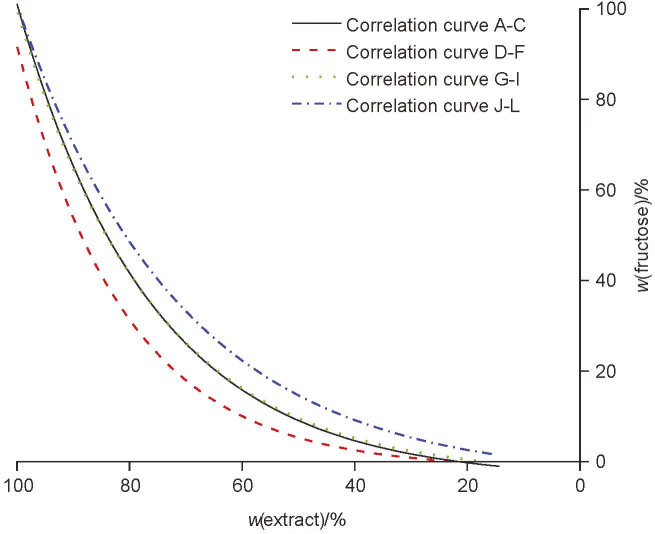
The correlation curves for fructose consumption with successive use of yeast starter culture *Saccharomyces pastorianus* in all four groups: curve A-C group 1, curve D-F group 2, curve G-I group 3 and curve J-L group 4

**Fig. S2:**
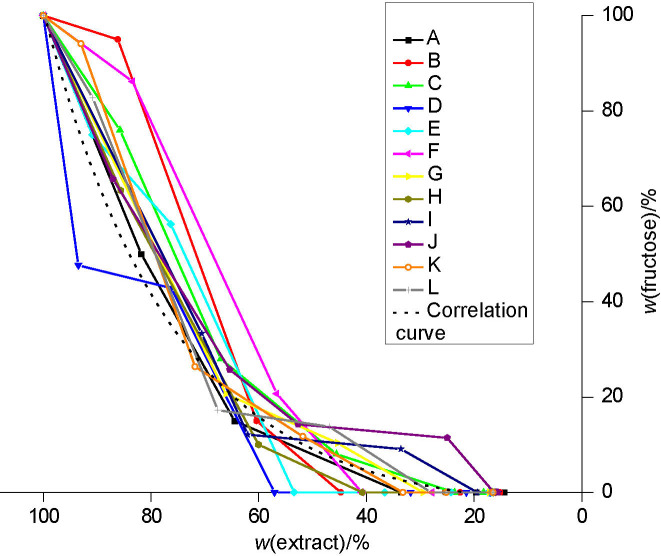
The fructose mass fraction in relation to the extract mass fraction during beer production with a successively used yeast starter culture (from the first (A) to the last (L) successive use)

The uptake of fructose during beer production with successive use of a starter *S. pastorianus* culture is shown in Fig. S2. According to the correlation curves shown in Fig. 4, the consumption of fructose was most effective when D, E and F yeast starter cultures (group 2) were used. The least efficient fructose consumption during the primary fermentation occurred when J, K and L yeast starter cultures (group 4) were used. During these primary fermentations, the glucose available for uptake was quickly exhausted. This confirms that glucose is the preferred substrate over fructose and could have an inhibitory effect on the uptake of fructose by *S. pastorianus* (*21*).

### Metabolism of DP2 saccharides

**Fig. 5:**
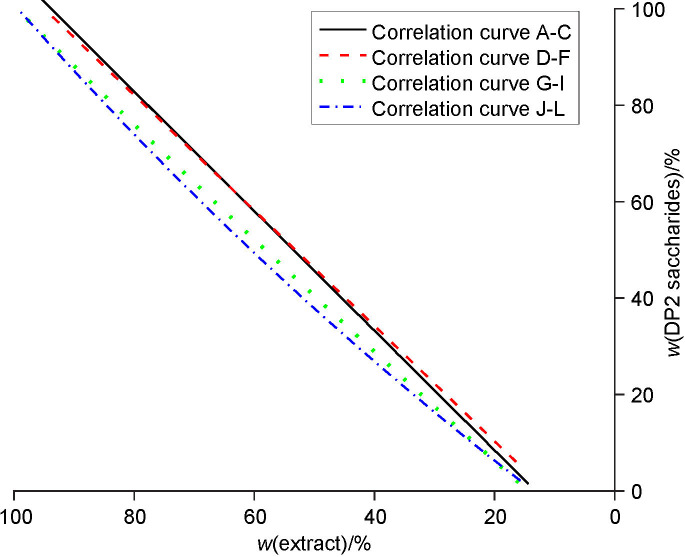
The correlation curves for DP2 sugar consumption with successive use of yeast starter culture *Saccharomyces pastorianus* for all four groups: curve A-C group 1, curve D-F group 2, curve G-I group 3 and curve J-L group 4

**Fig. S3:**
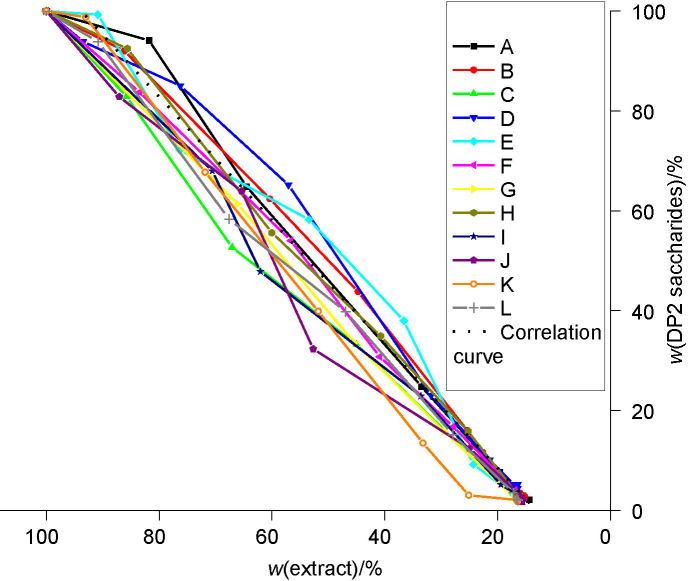
The DP2 saccharide mass fraction in relation to the extract mass fraction during beer production with a successively used yeast starter culture (from the first (A) to the last (L) successive use)

The consumption of DP2 saccharides during the primary and secondary fermentations is shown in Fig. S3. The consumption efficiency of maltose was lower at the beginning of the successive application of a single yeast culture (Fig. 5). The consumption of DP2 saccharides was more efficient with the successive use of the yeast starter culture in groups 3 and 4.

### Metabolism of DP3 saccharides

**Fig. 6:**
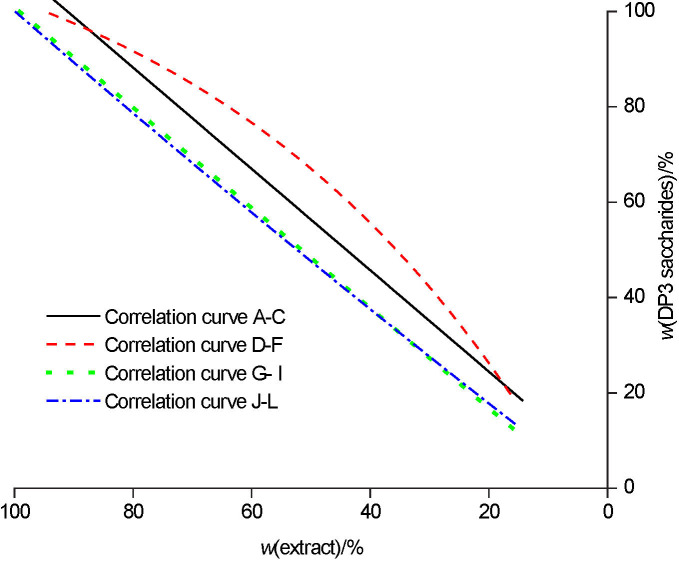
The correlation curves for DP3 sugar consumption with successive use of yeast starter culture *Saccharomyces pastorianus* for all four groups: curve A-C group 1, curve D-F group 2, curve G-I group 3 and curve J-L group 4

**Fig. S4:**
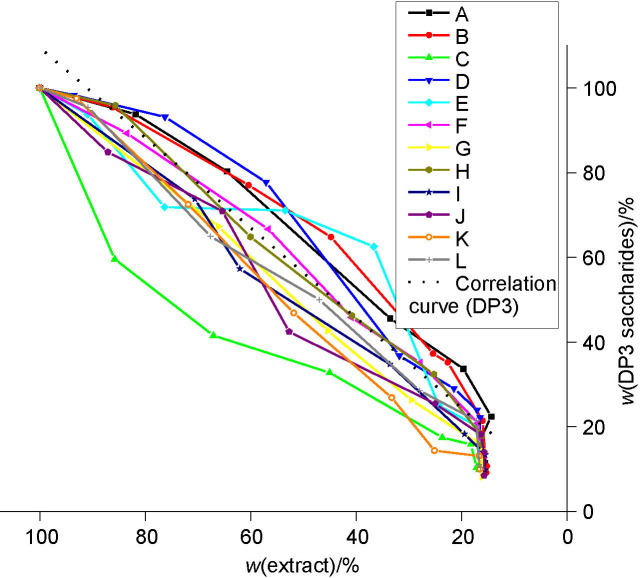
The DP3 saccharide mass fraction in relation to the extract content mass fraction during the beer production with successively used yeast starter culture (from the first (A) to the last (L) successive use)

The efficiency of DP3 saccharide (maltotriose) uptake during the fermentations with successive use of *S. pastorianus* starter culture was the most variable and is presented in Fig. S4. Fig. 6 shows the correlation curves for the DP3 saccharide consumption during beer production in all the four groups. The most unequal uptake of DP3 saccharides occurred in group 2, which included the successive use of the starter cultures of yeasts E, F and G. The most effective uptake of DP3 saccharides occurred in groups 3 (G, H and I fermentations) and 4 (J, K and L fermentations), in accordance with the DP2 consumption efficiency.

### Beer analyses

Beer quality is a complex issue, which includes several groupings of parameters: chemical, microbiological and sensory. All the beer that was produced for the purpose of this study was adequate in terms of the quality standards for lager beer (data not shown).

Table 2 shows the concentrations of DP2 and DP3 saccharides in the final product (from beer A to beer L). Because of effective fermentation, we were unable to detect glucose and fructose, since the concentrations of both were below the limit of detection (LOD=0.01 g/100 mL). The concentration of DP2 saccharides in beer was less than 0.2 g/100 mL, while of DP3 saccharides was less than 0.3 g/100 mL. The beer samples with the minimum residual saccharide concentration were I, J, K and L, which were produced by the last four successive uses of the yeast starter cultures. The quality parameter results indicated that all the final products obtained by the successive use of *S. pastorianus* starter culture were of adequate quality.

**Table 2:** The concentration of saccharides with degrees of polymerisation 2 and 3 (DP2 and DP3) in the final product (from beer A to L) produced with successive use of yeast starter culture *Saccharomyces pastorianus*

Final product (beer)	*m*(DP2)/*V*(wort)/(g/100 mL)	*m*(DP3)/*V*(wort)/(g/100 mL)
A	0.12±0.01	0.20±0.01
B	0.14±0.01	0.21±0.01
C	0.15±0.01	0.20±0.01
D	0.18±0.01	0.26±0.01
E	0.12±0.01	0.21±0.01
F	0.19±0.01	0.29±0.01
G	0.14±0.01	0.24±0.01
H	0.14±0.01	0.23±0.01
I	0.10±0.01	0.16±0.01
J	0.10±0.01	0.14±0.01
K	0.10±0.01	0.16±0.01
L	0.11±0.01	0.16±0.01

This study confirmed that the propagation of the starter culture in the same medium used in successive fermentations does not develop the maximum potential for yeast fermentation capacity and saccharide uptake efficiency (with the exception of fructose). As a consequence, yeast exploitation can be extended further. This was confirmed by the high viability of the yeast cells in all 12 successive uses of the *S. pastorianus* starter cultures. The microbiological conditions of the starter culture were also adequate, indicating good hygiene practices and proper handling of the yeast biomass during the rest period between repitchings (*25*). Jenkins *et al*. (*26*) showed that lager yeast viability and vitality was a function of the generation number. They observed that the impact of serial repitching on the yeast’s physiological state was not universal but instead was a strain-dependent phenomenon. Laboratory studies have shown that the general cellular viability decreased as the generation number increased in all studied lager yeast strains. However, this was not the case in this study, as the viability of our yeast starter culture remained high throughout all twelve successive fermentations (>95%).

This study focused on the impact of successive exploitation of a single *S. pastorianus* starter culture on saccharide uptake dynamics from wort and revealed an interesting dynamics of saccharide uptake. Namely, this indicates the improved uptake during the time course, and especially the uptake of DP2 and DP3 saccharides. The preferred uptake of glucose over fructose, DP2 and DP3 saccharides is well recognised. Nevertheless, the kinetics presented in Fig. 1 clearly shows that the uptake of glucose continues with fructose and is then followed by DP2 and DP3 saccharides. The kinetics shows the typical behaviour for enzymatic reactions of brewing yeast metabolism. Consequently, the decrease in the conversion rate detected during successive fermentations could be expected. Conversely, *S. pastorianus* under stable fermentation conditions shows improved conversion rates as a function of subsequent applications. A closer look at the yeast population structure shows that the first population has a ratio of young *vs* mature yeast cells that favours the younger population, which uses substantial energy for reproduction. By contrast, the older population grows in media that are more or less converted to the final product and the components are not consumed for reproduction, as reflected in the higher conversion rate and in attenuation of time.

## CONCLUSIONS

We conclude that the successive use of a single *Saccharomyces pastorianus* starter culture has an impact on the dynamics of saccharide utilisation from a classical hopped wort and young beer. The efficiency of saccharide consumption was proportional to the successive application of the single starter culture and was even enhanced, suggesting the possibility that successive fermentation can be continued since the yeast potential is fully developed after 12 cycles. The current technology and the availability of processes with higher hygiene standards would therefore appear to allow extension of the number of fermentations, because *S. pastorianus* appears to retain its biological and physiological capacity. It also remains healthy even after 12 successful exploitations of the yeast starter culture.
