# Corrigendum to: Impact of Successive Exploitation of a *Saccharomyces pastorianus* Starter Culture on Saccharide Uptake Dynamics from Wort (Published: Food Technol. Biotechnol. 59 (1) 16-23 (2021) https://doi.org/10.17113/ftb.59.01.21.6899)

**DOI:** 10.17113/ftb.59.01.21.6899_corrig.61(3)

**Published:** 2023-09

**Authors:** Miha Ocvirk, Nataša Kočar Mlinarič, Iztok Jože Košir

**Affiliations:** 1 Slovenian Institute of Hop Research and Brewing, C. Žalskega tabora 2, 3310 Žalec, Slovenia; 2 Pivovarna Laško Union d.o.o., Pivovarniška ulica 2, 1000 Ljubljana, Slovenia

The authors would like to state that they inadvertently omitted one of the authors when preparing the manuscript. The authors of this work are as follows:

Miha Ocvirk^1^, Nataša Kočar Mlinarič^2^, Peter Raspor^3^ and Iztok Jože Košir^1^*

^1^Slovenian Institute of Hop Research and Brewing, C. Žalskega tabora 2, 3310 Žalec, Slovenia

^2^Pivovarna Laško Union d.o.o., Pivovarniška ulica 2, 1000 Ljubljana, Slovenia

^3^Biotechnical Faculty, University of Ljubljana, Jamnikarjeva 101, 1000 Ljubljana, Slovenia

